# Quantification of Blood Flow in Internal Cerebral Artery by Optical Flow Method on Digital Subtraction Angiography in Comparison with Time-Of-Flight Magnetic Resonance Angiography

**DOI:** 10.1371/journal.pone.0054678

**Published:** 2013-01-24

**Authors:** Tzung-Chi Huang, Chih-Kai Chang, Chun-Han Liao, Yung-Jen Ho

**Affiliations:** 1 Department of Biomedical Imaging and Radiological Science, China Medical University, Taichung City, Taiwan; 2 Department of Radiology, China Medical University Hospital, Taichung City, Taiwan; University of Zurich, Switzerland

## Abstract

**Objective:**

This study compared data on the blood flow velocity in the internal carotid artery, which was obtained using the optical flow method (OFM) with digital subtraction angiography (DSA) and the time-of-flight (TOF) technique using magnetic resonance angiography (MRA).

**Materials and Methods:**

Images were obtained from 12 cerebrovascular patients who underwent both brain DSA and MRA imaging. The OFM was applied on the DSA images to determine the average blood flow velocity. The calculated results were compared with the values obtained from the TOF-MRA data. A linear fit was performed on the data and Bland-Altman plots were analyzed.

**Results:**

The blood flow velocity was closely associated with vascular diseases. Color-coding of the OFM measurements were superimposed on to the DSA images, which quantitatively illustrated the relative flow in the vessels. The average blood flow velocity was calculated using OFM and DSA, which demonstrated a high correlation with the MRA measurements in the anterior-posterior (AP) view (R = 0.71). In contrast, the average blood flow velocity was low in the lateral view (R = 0.28). The consistency between the high and low blood velocity in the AP view was better compared to the lateral view. The blood flow velocity distribution in the AP view was statistically closer to the MRA measurement compared to the lateral view.

**Conclusions:**

This study evaluated the correlation of blood flow measured using DSA and TOF-MRA in a small heterogeneous group of patients with cerebrovascular lesions. OFM with DSA imaging reveals hemodynamic information and TOF-MRA.

## Introduction

Hemodynamic information plays an important role in the visualization of anatomical structures and provides critical information for the diagnosis of head and neck vascular disease, treatment planning and evaluation [1]–[2]. Currently, there are several approaches to obtain the blood flow velocity in the head and neck blood vessels, including computed tomography (CT) perfusion, magnetic resonance (MR) perfusion, single-photon emission computed tomography (SPECT) and Doppler ultrasound. However, none of these techniques fully achieves the clinical requirements [3]–[5]. For example, a CT perfusion cannot cover the entire brain [6]–[7], a MR perfusion only provides semi-quantitative information [8]–[10], a Doppler ultrasound provides poor spatial resolution with significant signal attenuation through the skull and SPECT is not usually well-tolerated by the patients caused of radiation dose and long acquisition time [11]. Clinically, digital subtraction angiography (DSA) is more widely accepted in the diagnosis of neurovascular pathology [12]–[13]. Moreover, the syngo iFlow allows for a dynamic flow evaluation with visualization in a complete run in full color, which enhances the temporal resolution in the DSA. Thus, the quantification of blood flow using DSA is achievable with further image processing.

The phase contrast (PC) technique is often used in magnetic resonance angiography (MRA) to measure the blood flow velocity [14]. This non-invasive and non-radioactive technique is effective in the screening of vascular lesions. However, its temporal resolution limits its ability to detect fast and complicated blood flow variations. In contrast with the PC technique, the time of flight (TOF) technique has good temporal resolution and feasibility of blood flow velocity measurement [15]–[17].

In clinical diagnosis, conventional DSA only assesses hemodynamics on the sequential opacification of vascular structures using gray scales; however, the quantification of blood flow using DSA was absent. The optical flow method (OFM) is an image intensity gradient-based registration method, which calculates the small displacements between the corresponding pixels of two images on the basis of the image intensity patterns [18]. The relative blood flow velocity can be calculated using OFM in consecutive DSA images. Due to the good spatial and temporal resolution in DSA images, the application of the OFM on these images provides useful relative blood flow velocity estimations for clinical evaluations.

In this study, imaging data were obtained from 12 cerebrovascular patients who had DSA and MRA imaging performed on the internal carotid artery (ICA). The OFM was applied on the DSA images to calculate the relative blood flow velocity and were compared with the TOF-MRA measurements for a correlation analysis. Visualization of the calculated velocity using color coding was performed on the DSA images. With the add-on quantitative information of the flow, DSA not only provided images with high spatial and temporal resolution but also hemodynamic information. The latter was qualitative and based on observations made by the physicians, and was dependent on the physician’s personal experience and skills. Using this proposed technique, the hemodynamic information is objective and parametric.

## Materials and Methods

### Patient Data

Between September 2011 and August 2012, twelve cerebrovascular patients, seven males and five females with an average age 42 years, underwent DSA and MRA. Of these patients, three patients had an aneurysm, two patients had arteriovenous fistula and seven had arteriovenous malformations (AVM). The clinical patient characteristics are listed in Table 1. The participants provided their verbal informed consent to participate in this study. Because there was no extra step or conflict in the original imaging procedures for the patients and the patient’s personal names were removed prior to image processing, a written consent was not required. Two patients who enrolled in this study were minors and their parents/guardians provided their verbal consent for participation in the study. In this study, all of the information collected on the recruited patients was recorded on their clinical charts. The ethical committee agreed to use the verbal consent of the subjects in this study and the collection of clinical patient data was approved by the ethics committees of China Medical University Hospital, Taiwan (DMR100-IRB-181).

**Table 1:** The clinical patient characters.

Patient #	Sex	Age	
**1**	F	48	Left ICA aneurysm
**2**	F	50	Left ICA AVF
**3**	M	46	Right parietal AVM
**4**	F	48	Left ICA aneurysm
**5**	M	55	Right frontal AVM
**6**	M	11	Right frontal AVM
**7**	M	38	Left temporal AVM
**8**	M	33	Right temporal AVM
**9**	M	35	Right parietal AVM
**10**	F	65	Left ICA aneurysm
**11**	M	15	Right parietal AVM
**12**	F	69	Right ICA AVF

### Image Acquisition

A 4F (French unit) catheter was inserted into the ICA at the C1–C2 cervical vertebrae. A 70% diluted contrast agent (OMNIPAQUE 350 mg iodine/mL) (6 mL in volume) was injected into the artery in 1.5 seconds by an auto-injector (Liebel Flarsheim 903300 D). A biplane neuro X-ray system (Philips Allura Xper FD20/20) was used to take the DSA images with 6 frames/s in the anterior-posterior (AP) and lateral directions. The contrast entered the artery and filled the vessels at 4–7 frames in the DSA, which was used to estimate the blood flow average velocity using OFM.

Two magnetic resonance imaging instruments (General Electric SignaHDxt 3.0T, General Electric SignaHDxt 1.5T) were used to acquire the patient MRA image. The high-resolution coil, which was specifically used in the TOF applications, collected the signals. The pulse sequence was the TOF with an echo time (TE) between 1.6 and 2.7 ms, repetition time (TR) between 35 and 40 ms and a flip angle (α) of 25°.

### Estimation of the Blood Flow Velocity

**Figure 1:**
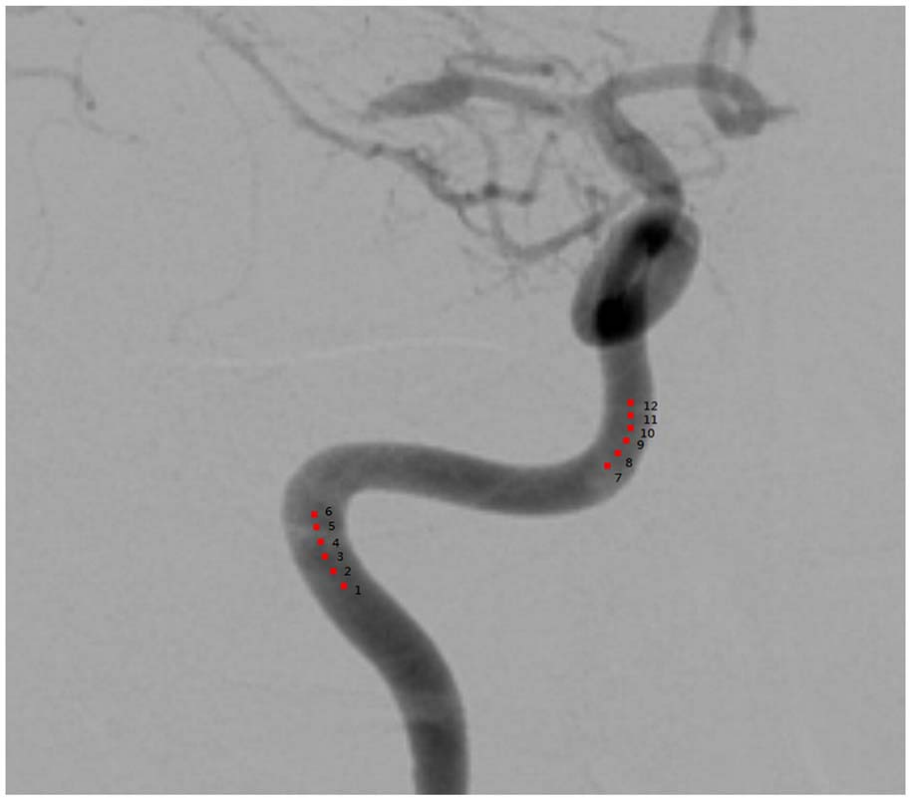
Selected ROI locations for flow estimation.

A blood flow velocity estimation system was developed in which the OFM was applied on to the DSA images to calculate the blood displacement between two consecutive images and blood velocity using the temporal resolution of the DSA images [18]. To increase the accuracy of the OFM, we added the “Iterative-Amendment Feature” to the original method. This modified optical flow process had been previously validated [19]. A total of 12 regions of interest (ROI) in addition to the ICA, consisting of 3 × 3 pixels, were selected to calculate the average velocity in the AP and lateral views, respectively. We used the average flow from the 3 x 3 surrounding pixels, instead of the single value calculated by the OFM, to represent the local flow estimation. The selected ROI locations for the flow estimation in the 12 cases are shown in Fig. 1. The calculated blood flow velocity distribution was visualized using color coding on the DSA images. The velocity matrix, which was calculated by the OFM, included the lateral and inferior-superior displacements, which corresponded to each voxel in the images. The spatial accuracy of the blood flow estimation, which was calculated by the OFM, was reported [19]–[20]. We previously applied the OFM to measure the relative velocities of blood flow using angiography and investigated the vascular effects on hepatocellular carcinoma patients who underwent transarterial chemoembolization [21]. The calculated velocity as determined by the OFM was compared with the Doppler ultrasound measurement and the results were consistent [21]. We also performed an optical flow analysis on the DSA to illustrate the potential calculation of intracranial blood flow in patients with cerebral vascular disorders and its therapeutic effects [22]. The OFM calculation equation is shown below 
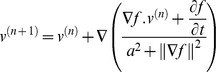
where n is the number of iterations, which was 100 in all of the estimations and 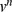 is the average velocity driven by the surrounding voxels. Moreover, *f* is the image intensity and α is the weight factor in which the value was empirically set at 5 for the DSA images [19].

TOF is the most commonly used technique in clinical blood vessel examinations. It is a flow-related enhancement (FRE) technique applied to blood flow imaging. When the blood flows into the plane where the signal is obtained, the hydrogen ions are not affected by the RF wave, which provide strong signals, while the surrounding static tissues are affected by the continuous RF wave and are saturated, thus emitting small signals. This difference is reflected in the MR images by different gray levels, which can easily distinguish the blood from the surrounding static tissues. According to these reports, when the flow velocity is less than 40 cm/s, the flow is faster; when the FRE is stronger, the signal is stronger. When the velocity is even higher, the signal TOF strength is lower [15]–[16]. The intensity of the TOF-MRA image inside of the ICA is directly used to estimate the relative blood flow velocity. Similar to the DSA and OFM methods, a total of 12 ROIs in addition to the ICA, consisting of 3 × 3 pixels, were selected for the average gray level values in the AP and lateral views.

### Statistic Analysis

In this study, the gray level values in the ICA obtained from the MRA images were treated as the reference of the blood flow velocity. The relative velocity values were calculated using OFM and DSA, which was then linearly fitted to the reference values using the MRA images. The correlation was analyzed on the basis of the fitted data. Linear regression equations were applied to convert the quantitative data from the OFM into TOF-MRA. A Bland-Altman plot was used to compare the consistency of the data. The box plot was applied to determine the relationship between the aneurysm, arteriovenous fistula, arteriovenous malformation and blood flow velocity in the ICA. All of the statistic analysis was performed using SPSS 16.0 (IBM SPSS Statistics; IBM Corporation, Armonk, NY, USA).

## Results

**Figure 2:**
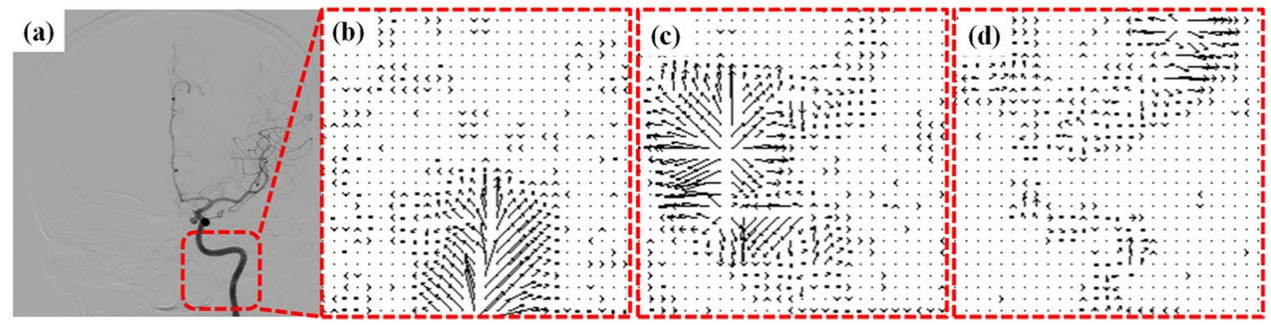
Visualization of the OFM calculation in the blood flow vectors. (a) The original DSA image. The red square represents the region of interest, which shows the visualization. (b–d) shows the blood flow vectors of the initial 3 consecutive time intervals when the contrast agent was injected into the ICA. The time interval was 1/6 s.

**Figure 3:**
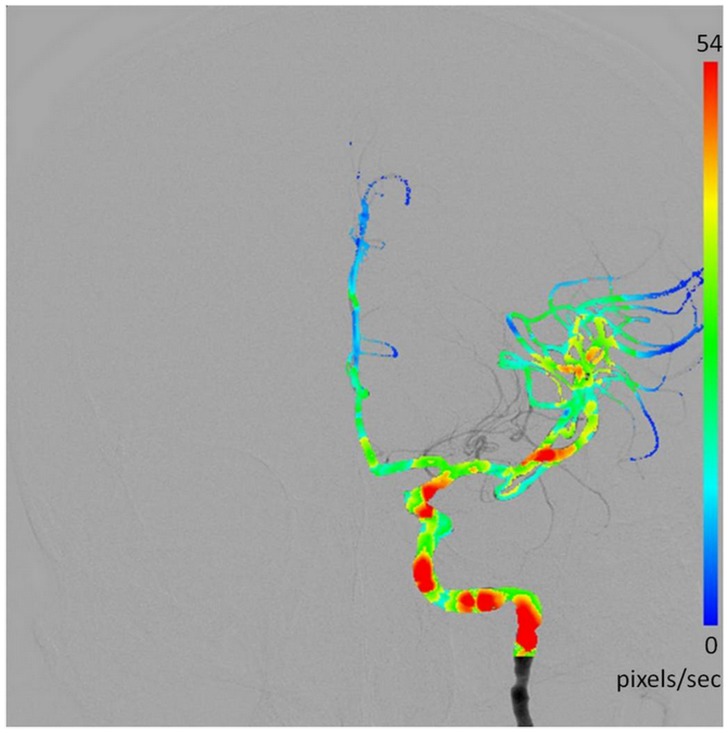
A representative OFM calculated blood flow velocity distribution is presented on the DSA images with color-coding in an aneurysm patient. The fast flow is in red and the slow flow is in blue. The maximum velocity was 54 pixels/second (red). (a) AP view; (b) lateral view.

**Figure 4:**
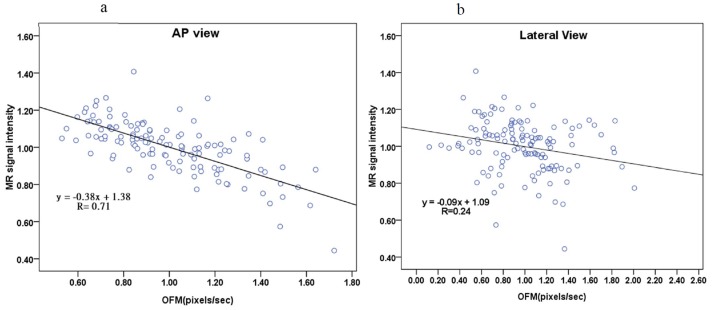
The linear fit between the OFM calculated blood flow velocity values and corresponding MRA measured signal intensity values. (a) AP view, R = 0.71; (b) lateral view, R = 0.24.

**Figure 5:**
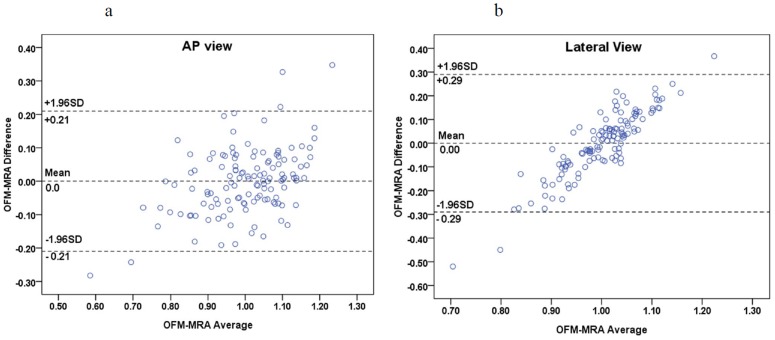
A Bland-Altman plot of the OFM calculations and MRA measurements: (a) AP view; (b) lateral view.

**Figure 6:**
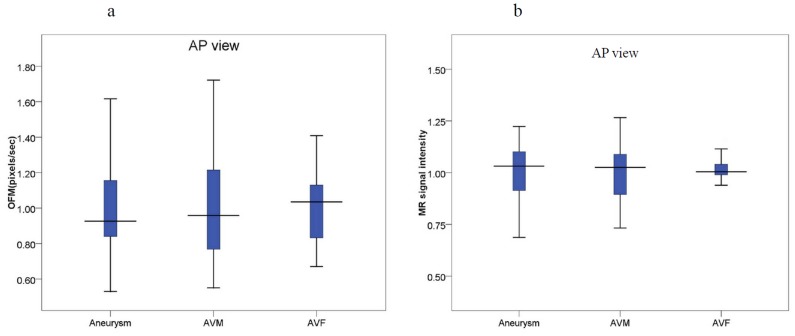
The box plots for an aneurysm, AVM, and AVF compared to (a) the blood flow distribution parameters in the AP view and (b) MRA measurement.

An example of the visualization of the OFM calculated blood flow variation is shown in Fig. 2. The original DSA image (Fig. 2(a–d)) demonstrates the flow vector field variation for the 3 consecutive DSA images with 1/6 s intervals. The displacement magnitude and direction are clearly illustrated in this figure. The displacement was divided by the time interval to obtain the velocity. The calculated flow velocity distribution was visualized using color-coding in the DSA images (Fig. 3). The color coding was then superimposed on to the DSA image to illustrate the relative flow value, which was quantitatively determined using OFM (e.g., the red color on the image and color bar of Fig. 3), which quantitatively indicates the relatively high flow in the vessel. The regions of fast blood flow are colored in red and the slow flow regions are colored in blue. The correlation obtained from the OFM was calculated from the velocity values, and the MRA gray level values for the blood flow velocity distribution in ICA were within the expected estimations. As shown in Fig. 4(a), the correlation for the AP view was high (R = 0.71), while the correlation for the lateral view was low (R = 0.24) (Fig. 4(b)). The consistency between the values of the OFM calculated velocity and MRA gray level was high in the AP view. The difference was uniformly distributed and the 95% confidence interval was narrow (Fig. 5(a)). In contrast, the difference in the lateral view varied with the velocity and the 95% confidence interval was wide (Fig. 5(b)). The box plots of the diseases compared to the blood flow measurements are shown in Fig. 6. The AP view in the OFM-DSA and TOF-MRA data showed that the aneurysm had a range of flow velocities (in pixel/s: OFM: 0.53–1.62, 1.0±0.26; MRA: 0.69–1.22, 1.0±0.14); for AVF, the OFM-DSA measurement was from 0.69 to 1.41 pixel/s with 1.0±0.20 pixel/s and TOF-MRA measurement was from 0.89–1.14 with 1.02±0.06 pixel; AVM was 0.55–1.72 with 0.99±0.28 pixel/s for OFM-DSA and was 0.73–1.27 with 1.01±0.12 pixel for the MRA.

## Discussion

Blood flow velocity is closely associated with vascular disease. Blood flow information helps not only in the diagnosis of vascular disease but also in the selection of the method of treatment [23]–[25]. Recently, Serafin *et al.* investigated that the diagnostic performance of 2-D DSA, 3-D DSA and TOF-MRA can successfully assess hemodynamics in AVM with validation using DSA [26]–[27]. Moreover, Yu *et al.* reported that 4-D dynamic MRA produced similar results. These two earlier studies had applied conventional DSA to assess hemodynamics on the sequential opacification of vascular structures using gray scales. However, the interpretation of these studies was qualitative and not quantitative and was based on the physician’s experiences and observations. We proposed that the OFM utilizes the available temporal information within the DSA to quantitatively determine blood flow and reveal a strong association between the TOF-MRA and OFM measurements, which confirms that OFM-DSA is able to provide hemodynamic information as well as TOF-MRA. Thus, the application of OFM in the AP view of the DSA images can successfully estimate the blood flow velocity and flow direction. Moreover, the velocity distribution in the blood vessels is clearly visualized using color-coding.

This study evaluated the OFM calculated blood flow velocity in the ICA by comparing the values to the gray level values as measured using TOF-MRA. In addition, the blood flow velocity distribution was analyzed in relation to neurovascular diseases. The average blood flow velocity was calculated using the OFM in the AP and lateral views in the DSA images. The high correlation between the calculations and MRA measurements in the AP view, and the low correlation in the lateral view, are consistent with previous studies [22]. The correlation between the average blood flow velocity and size of the ICA in the AP view was higher compared to the lateral view. The consistency in the AP view was also higher compared to the lateral view. The blood flow velocity difference between the OFM calculations and MRA measurements was uniformly distributed compared to the velocity value. In contrast, the difference was smallest in the median velocity value and increases in the velocity value, which increased or decreased from the median value, was observe din the lateral view. The OFM calculated velocity distributions compared to the disease are presented using box plots, which showed consistency with the MRA data measurements in the AP view compared to the lateral view. The differences between the AP and lateral views were caused by the direction in blood flow in the ICA. There were potentially additional flow sections where the flow direction was perpendicular to the imaging plane in the lateral view compared to the AP view, which caused inaccuracies in the velocity calculation by OFM in the lateral view. In previous studies using simulations, the accuracy of the OFM was acceptable [28]. However, in this study, the OFM was applied to the clinical DSA images in which the shape of the blood vessels and imaging parameters varied among the patients. These variations caused an accurate discrepancy between the simulation and clinical data. The results of this study confirmed the feasibility of using OFM with a clinical AP view of the DSA images to obtain additional hemodynamic information, which could be helpful in disease diagnosis.

There were other limitations in this study, which deserve attention. The significant consistency (almost all of the values were under a 95% confidence interval) between the TOF-MRA and OFM-DSA in the “AP” direction is shown in Fig. 5a. However, the correlation and consistent examinations for the TOF-MRA compared to the “lateral” data of the OFM-DSA were inadequate (Fig. 5b). Analysis on the lateral view of the DSA (Fig. 4b and 5b) demonstrated neither a consistency nor a correlation between the two methods. The OFM-DSA was limited in the detection of flow measurements in the lateral view of the DSA. Furthermore, the optical flow was defined as the distribution in the apparent velocities of movement in the brightness patterns between the images. Application of the “smoothness constraint,” which is the motion component in the direction of the local gradient in the image intensity function, was estimated [18]. Thus, when the flow was in turbulent or collateral circulation, the flow estimation by the OFM resulted in inaccurate calculations.

### Conclusions

This study correlated the blood flow measured by DSA and TOF-MR in a small heterogeneous group of patients with cerebrovascular lesions. OFM with DSA imaging reveals hemodynamic information as well as TOF-MRA.
